# The Advantages of Combining Therapies in Treating Psychiatric Patients

**DOI:** 10.3390/brainsci14070708

**Published:** 2024-07-15

**Authors:** Ravi Philip Rajkumar

**Affiliations:** Department of Psychiatry, Jawaharlal Institute of Postgraduate Medical Education and Research (JIPMER), Pondicherry 605006, India; jd0422@jipmer.ac.in

## 1. Introduction

Mental illnesses are among the leading causes of morbidity and disability worldwide, and the burden associated with these disorders has increased steadily over the past three decades [1]. The costs of these disorders extend not only to their direct effects on quality of life and functioning but to their associations with medical illnesses and premature mortality [2]. Mental illnesses are often underdiagnosed due to sociocultural and economic factors, such as the stigmatization of people with mental illness and a lack of access to mental health care [3]. Even when a given disorder is identified and treated, the response to standard forms of treatment is often incomplete. For example, only about 50–60% of patients with depression who are treated with an antidepressant improve symptomatically and only 30–40% remit completely [4]. Similar results (≈50% response and 33% remission) have been observed with psychotherapies for depression [5]. Due to the complex and poorly understood etiology of most mental disorders, the process of discovering novel treatments is both lengthy and challenging [6]. As a result, there has been considerable interest in the possibility of improving treatment outcomes by combining two or more existing treatments in the hope that they will act synergistically. Combined therapies have been shown to result in significantly better short- and medium-term treatment outcomes in several conditions, including depression, bipolar disorder, and obsessive-compulsive disorder [7,8,9]. Based on this evidence, some forms of combined therapy—for example, antidepressants plus cognitive behavioral therapy (CBT) or exposure and response prevention (ERP) for OCD [10], and mood stabilizers plus atypical antipsychotics for the acute and stabilization phases of bipolar mania [11]—have been incorporated into consensus guidelines and have become part of psychiatric practice. Other forms of combined therapy, though controversial or unsupported by the available evidence, are commonly used in real-world clinical practice, especially when patients do not respond to standard treatments [12]. The objective of the current Special Issue, “The Advantages of Combining Therapies in Treating Psychiatric Patients”, is two-fold:

- To provide an overview of the potential benefits of combined therapies in patients with various mental disorders.
- To examine variables, either biological or psychological, that may indicate a need for combined modes of treatment.

In doing so, it is important to recognize that “combined treatment” is not something that can be applied uniformly to all patients with a given mental disorder. Each patient with a given diagnosis, such as depression, is a unique individual with a specific clinical and psychosocial profile that may influence the need for such treatment or the response to it. Therefore, the best approach to combined treatment is one based on the principles of individualized or personalized medicine. The selection of specific pharmacological or psychosocial therapies should, as far as possible, be informed by the patient’s specific needs, risk factors, and even by genomic or other biological parameters insofar as there is robust evidence to support their use [13,14]. The papers included in this issue address this approach from multiple perspectives.

## 2. Specific Insights from Articles Published in This Issue

Adverse experiences in childhood, such as physical and emotional abuse, are associated with a poorer response to antidepressant medications [15]. In their evaluation of children and adolescents in Lodz Voivodship, Poland, Grzejszczak et al. (contribution 1) found that 3.5–6.4% experienced physical violence and 14.7–16.8% experienced emotional violence before and during the fourth wave of COVID-19. There was a slight decrease in the frequency of physical violence post-COVID-19, but older male adolescents were at a higher risk of violence at both time periods. Up to 25% of adolescents worldwide experienced significant symptoms of depression during the COVID-19 pandemic [16]. This study highlights the importance of enquiring about experiences of abuse in adolescents presenting with depression in a post-COVID-19 world and using this information to select the most effective treatment options. For example, those patients who do not respond to medication may benefit from the addition of trauma-focused CBT [17] or short-term psychodynamic psychotherapy [18].

Biological markers are increasingly being used to select both mono- and combined therapies in psychiatry. Testing for genetic variants affecting drug metabolism or molecular targets of drug action has been found to optimize antidepressant selection and improve treatment outcomes in patients with depression [19]. However, various other biomarkers, such as electrophysiological, neurocognitive, or biochemical parameters, may prove to be useful in selecting specific treatments [20]. Zouaoui et al. (contribution 2) conducted a meta-analysis of auditory steady-state responses (ASSRs), an electrophysiological marker of neural activity in auditory circuits, in patients with schizophrenia spectrum disorders. They found that 40 Hz ASSRs were significantly reduced in first-episode psychosis, moderately reduced in schizophrenia, and minimally altered in individuals at clinical or genetic risk for psychosis. They suggested that this parameter could be a potential biomarker of disease stage and response, particularly in relation to auditory hallucinations. This finding is of particular interest because it may be connected to an earlier report suggesting that a functional variant of the *DRD4* (D_4_ dopamine receptor) gene is associated with persistent auditory hallucinations and a poor response to antipsychotics [21]. It has been suggested that patients with this variant should receive combined therapy, such as CBT for auditory hallucinations. *DRD4* polymorphisms have been associated with altered neural electrophysiological parameters, including auditory evoked potentials, in healthy children and adults [22,23]. The possibility of a link between *DRD4*, altered ASSRs, and treatment response in schizophrenia is intriguing. In the future, it may be possible to use multi-modal biomarkers (such as a combination of *DRD4* genotype and 40 Hz ASSR measurements), either alone or in combination with clinical data, to guide the selection of appropriate treatment combinations for a given mental disorder [24,25]. 

Conceptual clarity and methodological rigor are required when identifying biomarkers of treatment response, particularly when combined therapies are involved. If studies are not designed in such a way as to address concerns such as bias, heterogeneity, statistical power, and confounding factors, it will not be possible to establish a meaningful link between a given marker and the effects of the treatment [26]. Rajkumar (contribution 3) reviewed the available evidence on possible biomarkers of response to adjunctive yoga-based interventions in patients already receiving serotonin reuptake inhibitors for depression. These treatments were associated with changes in peripheral levels of brain-derived neurotrophic factor (BDNF) and interleukin-6 (IL-6), but similar changes occurred in the control groups, and the included studies were not optimally designed to minimize bias. This review emphasizes the need for good study design when evaluating the biomarkers of response to combined therapies.

Controversies related to combined treatment in a psychiatric setting often arise in relation to polypharmacy, in which two or more psychotropic drugs are combined either to obtain an additive effect (“combination” in the literal sense”) or to potentiate the actions of an initial treatment that is only partially effective (“augmentation”). In general, polypharmacy is discouraged because of a limited evidence base and a substantial increase in the risk of adverse drug reactions and drug interactions [27]. However, some experts believe that polypharmacy can be a rational practice, combining elements of both art and science. It should not be used routinely but tailored to the needs of individual patients, particularly those who are non-responders or treatment-resistant [28]. In their review paper, Cipolla et al. (contribution 4) address the role of polypharmacy in a commonly encountered clinical scenario: treatment-resistant schizophrenia (TRS). At least 20–30% of patients with this illness do not respond to two or more trials of antipsychotics [29,30]. Clozapine monotherapy, which is the standard approach to TRS, is effective in only about 40% of patients and is associated with several potentially severe adverse effects [31]. Cipolla et al.’s findings suggest that a combination of long-acting injectable antipsychotics (LAI) is clinically feasible, potentially effective, and well-tolerated by patients with TRS. They also note that this data is largely based on case series and retrospective studies and emphasize the need for the replication of these results in prospective research, as well as the need to attend to long-term safety concerns. This approach may prove useful in patients who have not responded to a single LAI, who do not tolerate clozapine, or where treatment adherence is a concern [32]. 

A key component of combined therapy in psychiatry is an awareness of effective monotherapy options before deciding that a given patient requires more than one treatment. Many patients with schizophrenia experience depressive symptoms, and over 25% have severe symptoms associated with disability, impaired quality of life, and an elevated risk of suicide [33,34]. Such patients are often treated with a combination of an antipsychotic and an antidepressant, but only around 50–55% respond to this approach, and there is a significant risk of drug interactions [35]. Fiorillo et al. (contribution 5) review the efficacy of lurasidone, a novel antipsychotic with actions at several serotonin receptor subtypes, in alleviating these symptoms. They found that monotherapy with this drug was associated with significant reductions in depressive symptoms and was well-tolerated across several controlled clinical trials. These findings suggest that lurasidone, either alone or in combination with other antipsychotics, may be effective in patients with schizophrenia and prominent depression. This approach could be tried prior to the use of selective serotonin reuptake inhibitors (SSRIs) or other antidepressants.

Over the past three decades, several neuromodulation therapies, such as repetitive transcranial magnetic stimulation (rTMS), transcranial direct current stimulation (tDCS), deep brain stimulation (DBS), and vagal nerve stimulation (VNS), have proven to be effective in patients with specific mental disorders who do not respond to standard treatments. Neuromodulation therapies are generally used in combination with standard pharmaco- or psychotherapies [36,37]. VNS is an approved add-on therapy for treatment-resistant depression (TRD), with benefits sustained for up to two years and a good safety profile [38]. Kavakbasi et al. (contribution 6) evaluated multiple outcomes in a cohort of twenty patients with TRD. They found that VNS was associated with multiple benefits, including reduced depressive symptom severity, reduced numbers of hospitalizations, and a slight reduction in medication requirements, though only the first two were statistically significant. They also found that a history of response to electroconvulsive therapy (ECT) predicted better outcomes after VNS implantation. These findings confirm reports on the long-term benefits of VNS in TRD and illustrate how the rational use of combined therapies may reduce the burden associated with high-dose monotherapy. As past responses to ECT appeared to predict current responses to VNS, this report also raises the possibility of a general tendency to respond to neuromodulatory therapies that can be used to inform their selection. This remains to be confirmed in future prospective studies.

## 3. Future Directions

It is likely that the use of combined therapies in psychiatry will grow in complexity and applicability over time, particularly with the availability of a wider range of adjunctive treatment options. These include pharmacological agents, such as ketamine and psilocybin, novel neuromodulation protocols, and the availability of psychological therapies in an online mode, all of which could conceivably be combined in a patient [39,40]. Three examples will suffice to illustrate how far this field has come in the past decade. First, there is the recent approval of a combination of two drugs—dextromethorphan and bupropion, each acting through a distinct neurotransmitter pathway—for patients with depression [41]. Second, patients requiring psychotherapy are increasingly being offered a combination of in-person (“offline”) and online psychological interventions; this combination has been referred to as “blended care”. This is likely to become a focus of future research and debate, particularly if some or all of the online components are automated [42,43]. Third, the use of machine learning techniques on existing large datasets of patients with mental disorders—for example, from biobank or clinical trial samples—could identify a specific combination of clinical and biomarker variables that could predict the response to individual or combined treatments [44,45].

## 4. Conclusions

The papers included in this issue provide a bird’s-eye view of the many factors involved in the selection and administration of combined treatments in psychiatric settings. It is important that such treatments be used judiciously, keeping in mind the factors unique to each patient’s illness and life history, the safety profile of each treatment, and the possible interactions between them. At the same time, they should not be withheld from patients who have failed to respond to monotherapy or who might benefit from them. Combination therapies for mental disorders are an area of active research. It is likely that the next two decades will see the advent of an evidence-based, precision approach to the combined use of medications, psychotherapies, and neuromodulation strategies in psychiatry. At the same time, care must be taken to ensure that these treatment options are available and accessible to patients in low- and middle-income countries, who constitute the majority of those affected by mental illness.
